# Population genomics reveals a mismatch between management and biological units in green abalone (*Haliotis fulgens*)

**DOI:** 10.7717/peerj.9722

**Published:** 2020-08-19

**Authors:** Paulina Mejía-Ruíz, Ricardo Perez-Enriquez, Jorge Alberto Mares-Mayagoitia, Fausto Valenzuela-Quiñonez

**Affiliations:** 1 Centro de Investigaciones Biológicas del Noroeste S.C., La Paz, Baja California Sur, México; 2 CONACYT-Centro de Investigaciones Biológicas del Noroeste S.C., La Paz, Baja California Sur, México

**Keywords:** Population genomics, Fisheries management, *Haliotis fulgens*, ddRAD, SNPs, Green abalone, Abalone

## Abstract

Effective fishery management strategies should be based on stock delimitation and knowledge of the spatial scale at which species are distributed. However, a mismatch often occurs between biological and management units of fishery resources. The green abalone (*Haliotis fulgens*) supports an important artisanal fishery in the west coast of the Baja California Peninsula (BCP), Mexico, which has shown a declining tendency despite the several management measures. Thus, the aim of this study was to characterize the spatial patterns of neutral genomic variation of green abalone along the BCP to test whether the genomic structure patterns support the current green abalone management areas. To test this hypothesis, a set of 2,170 putative neutral single nucleotide polymorphisms discovered by a double digest restriction-site associated DNA approach was used on 10 locations along the BCP. The results revealed a population structure with three putative groups: Guadalupe Island and northern and southern BCP locations. The contemporary gene flow might be explained by local oceanographic features, where it is bidirectional within the southern region but with a predominant southward flow from the northern region. These findings indicated that the administrative areas did not match the biological units of *H. fulgens* fishery; hence, the stock assessment and management areas should be revised.

## Introduction

Effective fishery management includes stock delimitation and requires knowledge of the spatial scale of those biological units (Selkoe & Toonen, 2006; Hedgecock et al., 2007). Nevertheless, management units are predominantly based on administrative units (Bernatchez et al., 2017), and mismatches frequently occur between biological and management units (Reiss et al., 2009). This discrepancy might lead to a reduced productivity or the overexploitation of discrete subpopulations (Frank & Brickman, 2000; Kenchington, 2003; Hilborn et al., 2003). In the marine environment, determining the genetic structure and thus defining management units is challenging because marine organisms show both large effective population sizes and high gene flow. This situation results in a weak population differentiation where traditional genetic tools with a limited number of loci might have low power to define management units (DeWoody & Avise, 2000; Hauser & Carvalho, 2008; Allendorf, Hohenlohe & Luikart, 2010).

Novel genomic approaches enable discovering and genotyping a large number of loci in non-model species, which allow coping with previous technological limitations (Hammer-Hansen, Therkildsen & Pujolar, 2014) both increasing the power of determining accurate population differentiation (Luikart et al., 2003; Grewe et al., 2015) and improving the spatial resolution of population boundaries in several fisheries resources (e.g., *Thunnus alalunga*, Laconcha et al., 2015; *Salmo salar* L., Gilbey et al., 2016; *Thunnus albacares*, Pecoraro et al., 2018). Hence, incorporating genomic tools to fishery management will shed light to link biological and management units for long term fishery sustainability.

Most abalone fisheries have shown historical fluctuations in their biomass with a decreasing trend (Shepherd & Brown, 1993; Hobday, Tegner & Haaker, 2001; Dowling, Stephen & McGarvey, 2004; Appleyard, Carr & Elliott, 2009). Some of these fluctuations have been attributed to poor resource management, slow recovery potential of the species, overfishing, illegal fishing, diseases, and environmental factors (Hamm & Burton, 2000; Withler et al., 2001; Guzmán-del Próo et al., 2003; Der-Merwe et al., 2011). In Mexico, the green abalone (*Haliotis fulgens*) almost fully supports the artisanal abalone fishery along the Baja California Peninsula (BCP) (Diario Oficial de la Federación (DOF), 2018). Its distribution ranges from Point Conception, USA (34.5° N) to Bahia Magdalena, Baja California Sur, Mexico (25.5° N) (Guzmán-del Próo, 1992) and occurs from intertidal to ~24 m in depth. They are broadcast spawners that reach sexual maturity at a shell length of 6–12 cm (Sierra-Rodríguez et al., 2006). The reproductive season varies widely depending on the location since reproductive peaks have been reported in autumn–winter (Ortiz-Quintanilla & Leon-Carballo, 1988; Vélez-Arellano et al., 2017) and winter–spring (Vélez-Arellano et al., 2020); nevertheless, this aspect is not well understood since studies have focused mainly on a single location without considering all the distribution area. Larval development from laboratory is temperature-dependent, and the swimming phase may last from 4 to 15 days with an optimal temperature range from 20 °C to 23 °C (Leighton, 2000) that might limit long distance dispersal.

**Figure 1:**
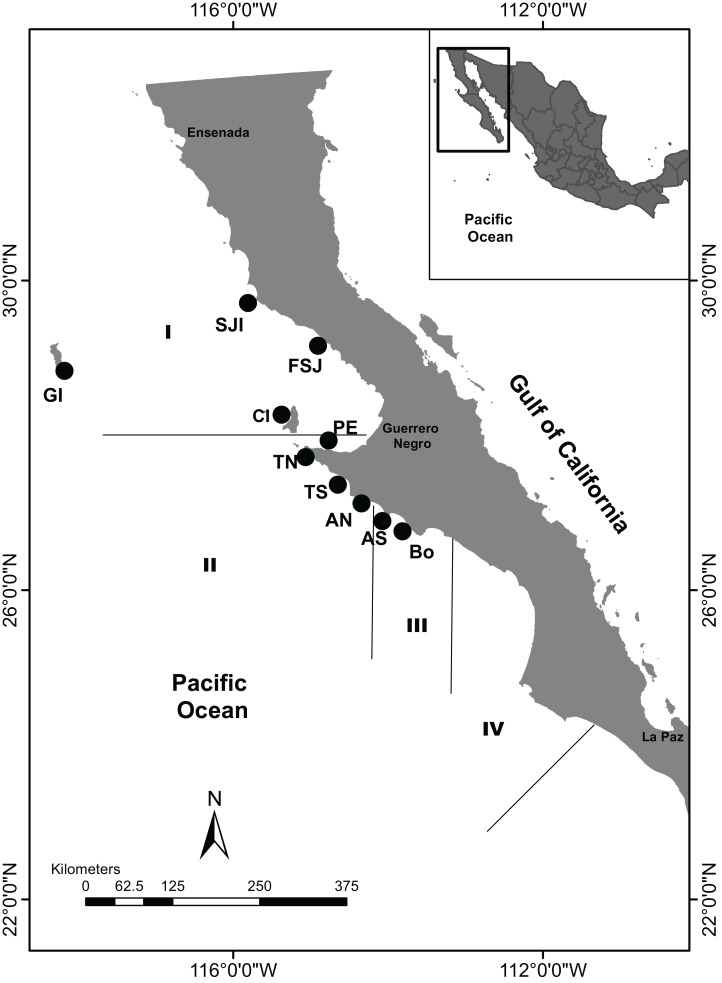
Sample sites along the Baja California Peninsula, Mexico. GI, Guadalupe Island; SJI, San Jerónimo Island; FSJ, Faro San José; CI, Cedros Island; PE, Punta Eugenia; TN, Tortugas North; TS, Tortugas South; AN, Asunción North; AS, Asunción South; Bo, Bocana. Black lines and Roman numerals indicate the four administrative zones of abalone fishery.

While the abalone fishing activity in the USA has been completely banned (California Department of Fish and Game Marine Region, 2005), in Mexico an extensive fishery occurs along the BCP. Although no management plan exists, the fishery has official regulations that considers four administrative zones (Fig. 1), which are established based on the latitudinal variation of life history (size at first maturity and reproductive season) (Diario Oficial de la Federación (DOF), 1993). Each zone has set a different legal minimum size and seasonal bans: zone I (150 mm, July 1–November 30); zone II (145 mm, August 1–December 31) and III (140 mm, August 1–December 31) and zone IV (120 mm, September 1–January 31) (Diario Oficial de la Federación (DOF), 1993, 1994; 2018). Additionally, a stock assessment has been performed by reef and cooperative concessions; since 2000 this regulation has mainly been focused on catch quotas assigned per fishing cooperative considering its annual growth rate (Sierra-Rodríguez et al., 2006). Despite all the regulation measures and significant effort by local fishermen to rebuild the stock(s), the abalone fishery is still listed as deteriorated with their populations below the optimum recovery level (Diario Oficial de la Federación (DOF), 2018).

Administrative zones are extensive areas in agreement with the latitudinal decreasing pattern in maturity size length from north to south along the BCP (Diario Oficial de la Federación (DOF), 1993; Sierra-Rodríguez et al., 2006) where abalone reef banks have shown a complex heterogeneity that might be related to habitat hydrodynamics and ecological features; as an alternate proposal, the fishery should be managed by reef groups of similar productivity (Guzmán-del Próo & Del Monte-Luna, 2017). However, the spatial scale administration of either current or proposed strategies (administrative zones, cooperative concession, reef groups, and separate reefs), remains as an undefined scale problem and requires the definition of biological units to support fishery management. In fact, the fishery management authority recognizes the need to define stocks as population units in well-defined polygons, with management strategies depending on the population status of each reef (Diario Oficial de la Federación (DOF), 2012). Therefore, management strategies may benefit from a genomic structure analysis using modern genomic techniques to provide insights about abalone populations across various spatial scales to support future stock assessment spatial scale and management actions.

The distribution range of *H. fulgens*—spanning ~10° in latitude—belongs to the temperate-tropical transition zone, characterized by a significant seasonal gradient influenced by the California and the North Equatorial Currents, with heterogeneous oceanographic and oceanic circulation conditions (Chelton, 1982; Lluch-Belda, 1999; Durazo et al., 2010; Durazo, 2015). The BCP can be divided into two major regions, with the headland of Punta Eugenia at 27.8° N as the limit between them. Punta Eugenia has previously been considered as a biogeographic break (Briggs, 1974) and evidence has shown that it could function as a gene flow barrier for some marine organisms (Bernardi & Talley, 2000; Terry, Bucciarelli & Bernardi, 2000; Bernardi, 2000; Huang & Bernardi, 2001; Muñiz-Salazar et al., 2005). A previous study using four microsatellite markers evaluated the genetic structure from eight coastal locations of the central-south region of the BCP and the oceanic Guadalupe Island (~250 km away from the coast) to assess population structure and define fishery stocks. The results showed no evidence of genetic differentiation among central-south locations, and low but significant genetic divergence between coastal locations and Guadalupe Island (Gutiérrez-González et al., 2007).

This study expands the previous work by collecting individuals from northern sites of the administrative Zone I and the use of a higher DNA resolution method using several thousand single nucleotide polymorphisms (SNPs) derived from double-digest RAD Sequencing (ddRAD-seq). Therefore, the aim of this study was to characterize the spatial patterns of neutral genomic variation among populations of the green abalone *H. fulgens*, following the hypothesis that population structure exists along the BCP, which does not match the current administrative fishery management areas. The use of more powerful neutral markers, such as SNPs and a wider range area should allow detecting a finer genetic structure than that previously reported. This study shows robust population structure patterns that should be considered to delineate stock boundaries, stock assessment models, and management actions to support fishery resilience.

## Materials and Methods

### Sample collection and study area

Collections were performed from July to August 2017 and May 2018 from 10 locations along the BCP, which belonged to three of four fishing administrative zones (Fig. 1). Abalones above the minimum legal size (*n* = 185) were collected under SAGARPA permits PRMN/DGOPA-011/2017 and PRMN/DGOPA/016-2018 by hookah diving during fishery evaluation surveys performed by the cooperative fishers. Epipodial tentacle fragments were taken without sacrificing the animals and preserved in 95% ethanol until DNA extraction (Table 1).

**Table 1:** Sampling locations and summary statistics averaged across 2,170 neutral SNPs among 10 localities of the green abalone (*Haliotis fulgens*).

Sample sites	Sample sites ID	Ns	Nsg	Ar	Ho	He	*F*_IS_*
Administrative Zone I							
Guadalupe Island	GI	20	20	1.850	0.272	0.282	0.020
San Jerónimo Island	SJI	13	13	1.847	0.274	0.278	0.003
Faro San José	FSJ	11	11	1.860	0.285	0.284	0.008
Cedros Island	CI	25	23	1.895	0.286	0.295	0.017
Administravie Zone II							
Punta Eugenia	PE	20	19	1.893	0.290	0.295	0.000
Tortugas North	TN	20	16	1.887	0.286	0.292	−0.005
Tortugas South	TS	20	20	1.893	0.292	0.296	0.007
Asunción North	AN	17	15	1.882	0.287	0.292	0.001
Administrative Zone III							
Asunción South	AS	19	19	1.881	0.284	0.291	0.006
Bocana	Bo	20	19	1.880	0.286	0.289	−0.019
Global		185	175	1.877	0.284	0.300	0.004

**Notes:**

Ns, Number of samples sequenced; Nsg, Number of samples successfully genotyped; Ar, Rarefied allelic richness; Ho, Observed heterozygosity; He, Expected heterozygosity; *F*_IS,_ Inbreeding coefficient.

* No *F*_IS_ value was statistically significant.

### DNA extraction and library preparation

Genomic DNA was extracted using a salt-extraction protocol (Sambrook, Fritsch & Maniatis, 1989) with RNase treatment. Genomic DNA integrity was verified using 1% agarose gels and quantified with a Qubit fluorometer 2.0 (Life Technologies, Carlsbad, CA, USA). All samples were normalized to a final concentration of 50 ng µl^−1^.

Double-digest restriction-associated DNA (ddRAD) libraries were prepared following Peterson et al. (2012) with some modifications; 500 ng of genomic DNA per sample was digested using the restriction enzymes EcoRI-HF (NEB^®^) and MspI (NEB^®^). A Pippin Prep device (Saga Science) was used to select fragment sizes from 376 bp–408 bp for all the libraries. Two ddRAD libraries were completed, each one with two pools; each pool comprised 48 individuals with a different barcode, and a unique index. A 4-index combinatorial and 48 barcodes described by Peterson et al. (2012) were used. To avoid batch effect, individuals from the same location were pooled in different pools and libraries in unequal proportions. Genotyping accuracy was validated using three replicates with different index-barcodes; then, the concordance genotype percentage between replicates of the same subject genotyped was assessed. The final libraries were sequenced on two lanes of an Illumina Hi-seq 4,000 platform at Novogene (Sacramento, CA, USA), using 150-bp pair-end reads.

### Raw data filtering and SNP calling

De-multiplexing and quality filtering were undertaken using the *process_radtags* pipeline in the STACKS software v1.30 (Catchen et al., 2011). Raw reads were trimmed to 140 base pairs to reduce sequencing errors present at the tail of the sequences. Read-pairs with the expected restriction sites and full barcodes at both ends were identified, allowing up to one error in each barcode. To homogenize the number of reads of each individual sample, they were standardized to 1 million reads each.

Reads were assembled de novo using the *denovo_map.pl* pipeline in STACKS. After several combinations of the main parameter optimization, the parameter combination was evaluated assessing the parameter effect and yield loci number as recommended in the literature (Paris, Stevens & Catchen, 2017; Rochette & Catchen, 2017) (Supplemental S1). The final parameter combination to build loci was a minimum stack depth of five (−*m* = 5), and a maximum of three nucleotide mismatches among stacks within individuals (−*M* = 3). To build the catalog, a maximum of four mismatches between sample loci between individuals (−*n* = 4) were allowed.

Further data filtering was performed using the population pipeline in STACKS, retaining those loci present in at least 80% of the individuals at each sampling site (−*r* = 0.8) in all of the localities 100% (−*p* = 10) and with a minor allele frequency MAF > 0.05. To prevent physically linked loci for subsequent analyses, the write_single_SNP option was used to retain only the first SNP from each RAD tag. Individual samples with >10% missing data across loci estimated with vcftools v.3.0 (Danecek et al., 2011), were excluded from subsequent analyses. The filtered vcf file was converted into the necessary file formats for downstream analyses using PGDSPIDER v.2.1.1.5 (Lischer & Excoffier, 2012). Linkage disequilibrium between all marker pairs was estimated using the function *pair.ia* of the package *poppr* v2.8.1 (Kamvar, Tabima & Grünwald, 2014) in R v.3.4.3 (R core Team, 2014) with 199 permutations; *p*-values were converted to false discovery rate (FDR) *q*-values (Benjamini & Hochberg, 1995) using the *p.adjust* function of the package *stats* v3.5.1 in R v.3.4.3 (R core Team, 2014) with a *q*-value of 0.05. Linked loci were discarded for further analysis.

### Outlier detection and Hardy Weinberg Equilibrium

To identify and eliminate potential candidate loci under selection, two independent methods were used. The first one employed a Bayesian approach to estimate the population specific *F*_ST_ coefficients using the BayeScan v3 software (Foll & Gaggiotti, 2008), which integrated population-specific (neutral genetic divergence) and locus-specific (selection) effects (Foll, 2012). The decision criterion to determine whether a locus was likely to be under a strong selection was the *q*-value (Foll, 2012) analogous to a FDR *p*-value that must be under 0.05. The BayeScan software was run with its default parameters set at a minimum number of 10,000 iterations, the length of 20 pilot runs to 5,000 iterations, and burn-in length to 50,000 iterations. For the second method, coalescent simulations were used to obtain a null distribution and confidence intervals around the observed values to see if the observed locus specific *F*_ST_ values could be considered as an outlier *F*_ST_, conditioned on the globallly observed *F*_ST_ value. For this purpose, Arlequin v3.5.2.2 software (Excofier & Lischer, 2010) was used with 1,000 simulated demes and 100,000 coalescent simulations were run to detect outlier loci based on their *F*_ST_ and *p*-value (*p* < 0.01).

Hardy–Weinberg equilibrium (HWE) was estimated with the software Arlequin v3.5.2.2 (Excofier & Lischer, 2010) by exact tests with 100,000 dememorization steps to remove those loci that were in disequilibrium in five (50%) or more locations (*p* < 2.3 × 10^−5^ after Bonferroni correction).

### Summary statistics

Genetic diversity estimates, such as allele frequencies, rarefied allelic richness (*Ar*), observed heterozygosity (*H*_*o*_), expected heterozygosity (*H*_*e*_) and Inbreeding coefficient (*F*_IS_) were estimated with the package *PopGenReport* v.3.0.4 (Adamack & Gruber, 2014) in R v.3.4.3 (R core Team, 2014). Kruskal–Wallis test and a posteriori Gao test were conducted to examine the differences between genetic diversity values of localities with the packages *stats* and *nparcomp* v.3.0 (Konietschke, Noguchi & Rubarth, 2019) in R v.3.4.3 (R core Team, 2014).

### Population structure analysis

The *F*_ST_ was calculated using the *basic.stats* function in the R package *hierfstat* v.0.04-22 (Goudet & Jombart, 2015). The differentiation degree between locality pairs was quantified using θ, an unbiased estimator of *F*_ST_ (Weir & Cockerham, 1984) in Arlequin v.3.5.2.2 software (Excofier & Lischer, 2010). The significance level was assessed using 10,000 permutations and corrected for multiple comparisons using the Bonferroni correction.

A discriminant analysis of principal components (DAPC) was performed in the R Package *adegent* v.2.1.1 (Jombart, Devillard & Balloux, 2010). This method maximize the variance among groups while minimizing the variation within groups and does not make any assumptions about the underlying population genetic model. To assess the optimal group number the function *find.clusters* was used with the Bayesian information criterion (BIC) method. To estimate the number of principal components (PCs) to be retained, the function *optim.a.score* was used.

A Bayesian clustering method with the software STRUCTURE v.2.3 (Pitchard, Stephens & Donnelly, 2000) was used to infer population structure and identify the most likely value of *K* (groups or putative populations), based on the admixture ancestry model and correlated allele frequencies. Runs consisted of an initial burn-in of 10,000 Markov Chain Monte Carlo (MCMC) iterations followed by 100,000 iterations for each inferred cluster (*K* 1 to 10 with five replicates) and the *locprior* model. The delta *K* method was used to determine an optimal *K* (Evanno, Regnaut & Goudet, 2005).

Analyses of molecular variance (AMOVA) using Arlequin v3.5.2.2 software (Excofier & Lischer, 2010) were performed with 10,000 permutations to evaluate the genetic variation within and between the different groups testing for several hierarchical hypothesized structures: (A) administrative zones as genetic groups; (B) two groups based on STRUCTURE results; (C) three groups based on *F*_ST_ results; (D) three groups based on DAPC and the unweighted pair group method with arithmetic mean (UPGMA) results; and (E) four groups based on visual division on the DAPC results.

An UPGMA tree was constructed using the program Phylip (Felsenstein, 1989) based on genetic distance estimates among populations (Cavalli-Sforza and Edwards chord distance, Dc) with 3,000 bootstrap replicates. The consensus tree was visualized using the Treeview software v1.6.5 (Page, 1996).

To further investigate the directional relative migration rates between localities, migration networks were generated using *divMigrate* function (Sundqvist et al., 2016) of the R package diveRsity (Keenan et al., 2013) with *G*_st_ (Nei, 1973) as a measure of genetic distance with 1,000 bootstrap repetitions and an arbitrary filter threshold of 0.60. Gene flow patterns were visualized using network graphics produced with the R package *qgraph* (Epskamp et al., 2012). Putative source and sink populations were identified by *divMigrate* as it calculates relative levels of migration by assessing the genetic differentiation between two populations and a hypothetical pool of migrants.

Finally, isolation by distance (IBD) was tested in the R Package *adegent* v2.1.1 (Jombart, Devillard & Balloux, 2010) using a Mantel test (Mantel, 1967) by correlating Slatkin’s linearized [*F*_ST_/(1 – *F*_ST_)] (Rousset, 1997) vs. geographical distance (shortest waterway distance in kilometers between sampling location pairs) with the function *mantel.randtest* using a Monte Carlo simulation with 999 permutations. These tests were performed at three hierarchical levels (1) including all locations; (2) using only coastal locations (excluding Guadalupe Island); and (3) within the central-south locations (excluding Guadalupe and San Jeronimo islands and Faro San José). To test whether the correlations between genetic and geographic distances were the result of a continuous or distant patchy cline of genetic differentiation, a 2-dimensional Kernel density estimator was applied with the function *kde2d* using the *MASS* package (Venables & Ripley, 2002) in R v.3.6.2 (R core Team, 2014).

## Results

### Individuals and single nucleotide polymorphism filtering

A total of 1,420,020,148 pair-end raw reads were obtained from two sequencing lanes. After applying the quality filters with the *process_radtags* program of STACKS (barcode or restriction enzyme site absence, low-quality reads), 96.8% of the reads were retained, resulting in an average of 7,458,559 reads per sample (range: 0.33 M–23.3 M). Individual samples with less than 1 million reads were discarded (*n* = 8). The remaining samples were standardized to 1 million reads and initial catalog of 116,443 RAD loci with 353,332 SNPs was built with the *denovo_map.pl* module in STACKS, from which the *populations* module extracted a dataset of 2,216 loci with an average read depth of 32X. Individuals with >10% of missing genotypes also were excluded (*n* = 2). After the FDR correction, no evidence was observed for significant linkage disequilibrium between loci (*p* > 0.05). which resulted in a global database of 175 individuals and 2,216 SNPs.

### Outlier detection and Hardy Weinberg equilibrium

Forty-five loci outliers were identified by Arlequin and five by Bayescan (five were common in both methods), leaving a dataset of 2,171 SNPs (see Supplemental S2 for details on the number of SNPs removed after applying quality filters). One locus showed significant deviations from HWE (*p* < 0.05) in more than half of the locations and it was removed from the database, leaving a neutral data set of 2,170 SNPs. Genotyping accuracy was evaluated using individual replicates, and an average genotyping error rate of 0.04% was recorded.

### Summary statistics

The estimates of global neutral genetic diversity were *H*_*o*_ = 0.284; *H*_*e*_ = 0.300; *Ar* = 1.877. Samples from the northern region (Guadalupe and San Jerónimo islands, and Faro San José) were the least diverse, while Punta Eugenia, Cedros island, and Tortugas South were the sites with the highest diversity (Table 1). Significant differences in genetic diversity values were found among localities, of which Guadalupe and San Jerónimo islands those with more pairwise significant differences (Supplemental S3). The *F*_IS_ was 0.004 (*p* = 0.38) and did not show significant values across locations (*F*_IS_ = −0.019 to 0.020; Table 1).

### Population structure analysis

The population substructure was significant (*F*_ST_ = 0.005, *p* = 0.006). Pairwise comparisons showed significant differences between the oceanic Guadalupe island (~250 km away from the coast) and the rest of the localities (*p* < 0.001). Aditionally, the northern coastal San Jerónimo island had significant *F*_ST_ estimates with all locations (*p* < 0.001) with the exception of Faro San José. Sites within the southern region showed no significant genetic divergence values among localities except for Faro San José with Tortugas South (Supplemental S4; *p* < 0.001).

**Figure 2:**
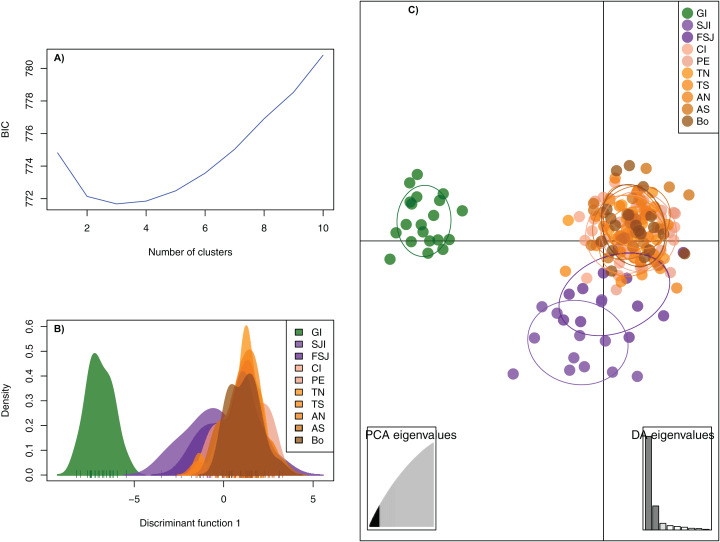
Discriminant Analysis of Principal Components (DAPC) based on 2,170 neutral SNPs of the green abalone (*Haliotis fulgens*). (A) Bayesian Information Criterion (BIC) values versus number of clusters (*K*). (B) Plots of population distributions based on the first discriminant function. (C) DAPC scatterplot: all DAPCs include a 95% inertia ellipsis for each population, with each dot representing a single individual. 28 PCs and three discriminant functions (dimensions) were retained during the analyses. Legends are ordered from north to south GI, Guadalupe Island; SJI, San Jerónimo Island; FSJ, Faro San José; CI, Cedros Island; PE, Punta Eugenia; TN, Tortugas North; TS, Tortugas South; AN, Asunción North; AS, Asunción South; Bo, Bocana.

**Figure 3:**
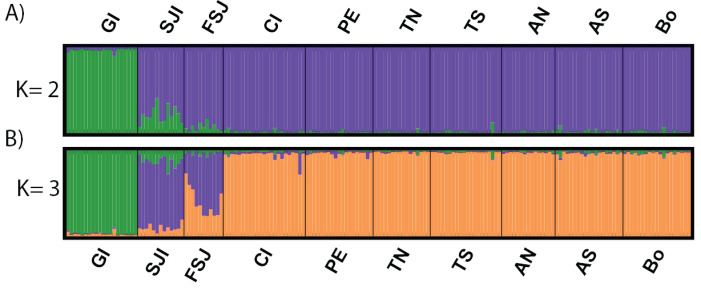
Results of individual clustering analysis implemented with STRUCTURE software based on 2,170 neutral SNPs among 10 sample sites for the green abalone (*Haliotis fulgens*). (A) *K* = 2 and (B) *K* = 3. Each vertical bar represents an individual and the color of each bar correspond to individual’s estimated membership of each of the clusters. Legends are ordered from north to south GI, Guadalupe Island; SJI, San Jerónimo Island; FSJ, Faro San José; CI, Cedros Island; PE, Punta Eugenia; TN, Tortugas North; TS, Tortugas South;, AN, Asunción North; AS, Asunción South; Bo, Bocana.

The DAPC analysis retained 28 principal components and identified three genetic clusters, one composed by the oceanic Guadalupe island, the second by the coastal northern locations (SJi and FSJ), and the third by coastal central-southern localities (CI, PE, TN,TS, AN, AS, and Bo) (Fig. 2). The Bayesian cluster analysis in STRUCTURE revealed two genetic clusters (*K* = 2) based on the delta *K* statistic of Evanno, grouping all individuals from Guadalupe Island separately from the rest of the coastal locations (Fig. 3A). However, a visual inspection of STRUCTURE admixture plots for *K* = 3 (Fig. 3B) showed an additional population subdivision resulting in a grouping pattern similar to the DAPC (Fig. 2) and the *F*_ST_ results (Supplemental S4).

Hierarchical AMOVA supported a regional population structure where only the grouping C (GI, SJI, all other localities), D (GI, SJI + FSJ, all other localities), and E (GI, SJI, FSJ, all other localities) resulted in a significant variation among groups (*F*_CT_ = 0.0104−0.0148; *p* < 0.05). However, only the last grouping (E) that consider Guadalupe Island, San José Island, Faro San José and the rest of the localities as separated groups, the differentiation among populations within groups (*F*_SC_ = 0.0005, *p* = 0.22) was no longer significant (Supplemental S5). Neither did any of these regional groupings support the current administrative zones (A) which resulted in no significant variation among groups (*F*_CT_ = 0.0003; *p* > 0.05) (Supplemental S5).

The UPGMA tree identified two to three major groups with a high bootstrap value (100%). The most robust topology separated the northern sample sites (Guadalupe and San Jerónimo island and Faro San José) from the rest (Supplemental S6).

### Gene flow analysis

**Figure 4:**
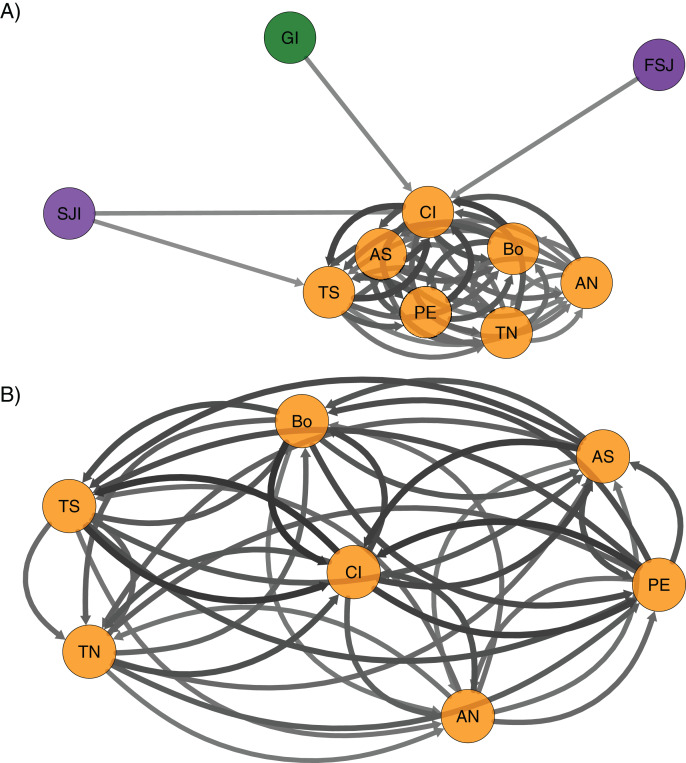
Relative migration networks for the green abalone (*Haliotis fulgens*) based on 2,170 neutral SNPs among 10 sample sites obtained using the Nei’s *G*_st_ based estimate in the package divMigrate. Only migration rates >0.6 are represented, circles represent localities, while arrows indicate the direction and magnitude of relative migration levels. Darker arrows indicate a stronger migration relationship compared to lighter arrows. (A) All the 10 localities and (B) southern region. GI, Guadalupe Island; SJI, San Jerónimo Island; FSJ, Faro San José; CI, Cedros Island; PE, Punta Eugenia; TN, Tortugas North; TS, Tortugas South; AN, Asunción North; AS, Asunción South; Bo, Bocana.

The directional relative migration using *divMigrate* (Fig. 4A) showed a predominant gene flow from northern locations (Guadalupe and San Jerónimo Island and Faro San José) to the rest of the southern sites. Despite the geographic proximity of San Jerónimo Island from Faro San José, migration between these locations was lower compared to the rest of the localities. A close examination of the connectivity among localities in the southern region showed no apparent predominant directional flow (Fig. 4B).

### Isolation by distance

**Figure 5:**
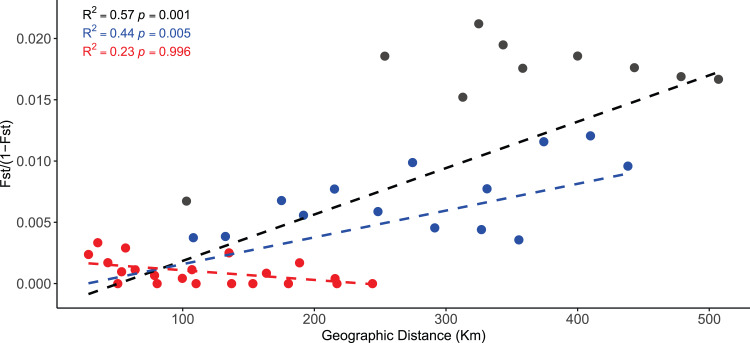
Isolation-by-distance (IBD) plots of linearized *F*_ST_ values against the lineal geographic distance of the green abalone (*Haliotis fulgens*) based on 2,170 neutral SNPs and 10 sample sites. Black line: IBD for all sample sites, blue line: IBD for 9 sample sites (without the most isolated location) (GI), and red line: IBD for 7 sample sites (central-south locations).

The Mantel test showed a strong relationship between linearized *F*_ST_ and geographical distances on the full data set (Mantel *r* = 0.76, *p* = 0.001) (Fig. 5). The regression analysis showed this relationship to be positive and linear (*R*^2^ = 0.57, *p* < 0.001). This pattern of IBD remains significant without the most isolated location, Guadalupe Island (Mantel *r* = 0.54, *p* < 0.017; *R*^2^ = 0.44, *p* = 0.005), but it was no longer significant when only the central-south locations were analyzed (Mantel *r* = 0.34, *p* = 0.129; *R*^2^ = 0.23, *p* = 0.996). This pattern was further explored using 2-dimensional kernel density plots. The dataset—including all locations—showed a discontinuity indicating a patched pattern of genetic differentiation among populations (Supplemental S7A). However, the other scatterplots showed a single patch (Supplemental S7B and S7C). These results support the regional green abalone population structure along the BCP.

## Discussion

The population structure results of green abalone along the BCP supports three regional genetic groups showing a clear differentiation between the oceanic island (Guadalupe Island, ~250 km apart from the coast) and the rest of the coastal localities; the second group is formed by the northern coastal localities, which include San Jerónimo Island and Faro San José; and the third group includes the southern coastal localities (Cedros Island, Punta Eugenia, Tortugas North, Tortugas South, Asunción North, Asunción South, and Bocana).

Differentiation between coastal localities and Guadalupe Island and panmixia along the central west coast of the BCP had already been reported (Zúñiga et al., 2000; Gutiérrez-González et al., 2007) supporting the results. This study expanded upon these early works covering a larger spatial scale for *H. fulgens* and it was able to detect a significant population structure between northern and southern coastal locations.

Gruenthal et al. (2013) reported patterns of genetic homogeneity for *H. fulgens* along the northernmost part of the species distribution (south of the California Bight) using a panel of 1,209 putative neutral SNPs. However, this previous study was performed in a reduced area of the species distribution range and differences might exist between the USA and Mexican locations, as studies have reported in *H. corrugata* using microsatellites (Díaz-Viloria et al., 2009; Munguía-Vega et al., 2015). *H. fulgens* seems to have an extensive gene flow among coastal locations, and the spatial scale of previous studies has been a factor to conclude panmixia. Based on the results, a sample compilation of the full species distribution range is necessary to capture a broader genomic structure and understand the biocomplexity of this fishery resource.

Shepherd et al. (1991) reported that *H. fulgens* juveniles and adults have limited movements, an average of ~14 min in a year (Shepherd et al., 1991). The main dispersal mechanism takes place during their pelagic larval stage, which can last from 4 to 15 days with an optimal temperature range from 20 to 23 °C (Leighton, 2000). Information about larval connectivity among local *H. fulgens* populations is scarce. Based on a drift tube experiment in the southern California Bight, *H. fulgens* larvae may move ~46 km downcoast in spring and 12 km upcoast in autumn (Tegner & Butler, 1985). Based on the regional hydrodynamics on the BCP central coast, the dispersal was also hypothesized to be limited and restricted to short distances because of the weakening of the California Current caused by kelp beds and the geographical complex coastline (Guzmán-del Proo et al., 2000). The results in this study suggested that the dispersal potential of *H. fulgens* might not be as limited as it has been assumed (Leighton, 2000; Morgan & Shepherd, 2006; Guzmán-del Proo et al., 2000) particularly in the central BCP.

Isolation by distance often shows biased results for cases of hierarchical structure (Meirmans, 2012). Mantel tests resulted in a significant relationship when considering all locations, even when using only coastal locations, but it was not significant within the coastal southern locations. Considering the separate clusters identified by STRUCTURE and DAPC, IBD seemed to be biased by regional population structure. Hence, IBD only plays an important role at a broad spatial scale in the system but not at regional level. This situation suggests a restricted gene flow among green abalones between the northern and southern regions and high levels of gene flow among the central and southern localities rather than a step-stone-structure.

In addition to the geographical distance, coastal features, such as headlands, generate convergences, eddy systems with counter currents, and stagnation zones, which may concentrate or retain larvae (Morgan & Shepherd, 2006). Along the BCP, the headland of Punta Eugenia has been proposed as a transitional biogeographic zone for tropical and temperate species limiting larval dispersion (Briggs, 1974; Hewitt, 1981; Lavaniegos, 2014). Moreover, this region has been considered as a phylogeographic break for many marine species (e.g., *Fundulus parvipinnis*, Bernardi & Talley, 2000; *Girella nigricans*, Terry, Bucciarelli & Bernardi, 2000; *Embiotoca jacksoni*, Bernardi, 2000; *Gillichthys mirabilis*, Huang & Bernardi, 2001). However, this genetic break is not limited to a narrow area and might cover a wider region northward (Haupt, Micheli & Palumbi, 2013). The regional partition detected for green abalone partially coincides with the region where those previous studies detected population divergence. Hence, the results suggest that the genetic differentiation between the north and south regions might be related to this biogeographic break. Nevertheless, further sampling is necessary to precisely determine the area where the coastal split of *H. fulgens* occurs along the coast. The underlying mechanism that maintains the regional partition might be related to the oceanographic features of the region and habitat availability that limits the connectivity among regions. Central-south BCP has a long continuum of rocky reefs; however, the region along the coast north of Punta Eugenia (Guerrero Negro to San Jeronimo) is an extensive region with sandy habitats (Terry, Bucciarelli & Bernardi, 2000) that are not suitable for green abalone. The asymmetrical contemporary flow showed a strong bidirectional gene flow within the southern region and a predominant southward flow from the northern-southern regions. These results are consistent with the local oceanographic features where the dynamics of the sea surface currents (<200 m) along the BCP are predominantly southward with a large influence of the California Current during the year in the northern region (Lynn & Simpson, 1987; Durazo, 2015). However, seasonal changes in the ocean circulation are favored in the southern region during summer and autumn (Lluch-Belda et al., 2000). A surface counter current has also been reported in shallow regions near the coast where these small-scale processes may have provided a physical mechanism that favored transport and larval recruitment (Durazo et al., 2010; Durazo, 2015). This mesoscale process in the southern region coincided with the spawning peaks (autumn and winter) reported for *H. fulgens* (Ortíz-Quintanilla & León-Carballo, 1996; Vélez-Arellano et al., 2017).

Therefore, larval transport (genetic flow) may have been promoted within the central and southern zones of the BCP. The seasonal oceanographic circulation also might enhance larval dispersal between regions during the spawning season when the California Current is more intense (winter) with a southward direction, but the large habitat discontinuity north of Punta Eugenia might have a crucial role limiting larval connectivity between regions. Nevertheless, a seascape approach is needed to test the effect of ocean currents in neutral green abalone population structure.

### Management implications

An essential prerequisite of a sustainable fishery management is the match of biologically meaningful entities and management units. Assessing population status or developing a management strategy might not be possible without first knowing where a population begins and ends (Funk et al., 2012). Unfortunately, fish stocks do not often correspond to true biological populations (Reiss et al., 2009), which may lead to reduced productivity, genetic diversity loss, local population reduction, and in extreme cases, to a local population collapse. The risk for such loss depends both on the structure itself and the size of the component populations (Laikre, Palm & Ryman, 2005). Hence, understanding biocomplexity within stocks is important to maintain their resilience to future environmental change (Hilborn et al., 2003).

The abalone fishery in the BCP has been regulated mainly by minimum legal size, closed seasons and zones, annual quotas, and internal controls of the cooperatives (Diario Oficial de la Federación (DOF), 1993, 1994). A contemporary stock assessment uses reefs as separate stocks, and since 2000, management has mainly used catch quotas, which are assigned by reefs considering an annual growth rate for each reef (Sierra-Rodríguez et al., 2006). Nevertheless, the separate management units that include only a portion of a larger population may lead to population stock dynamics bias (Mullins et al., 2018) and then of the reference points, as well as stock recovery times (Hilborn & Walters, 1992; Caddy & Mahon, 1995; Sierra-Rodríguez et al., 2006). This study addressed a need asked by the Mexican National Institute of Fisheries in assessing and characterizing the genetic composition of *H. fulgens*’ reefs within and between zones (Sierra-Rodríguez et al., 2006). The results suggest that *H. fulgens* biological units do not support the administrative areas (Fig. 1) of the abalone fishery, so stock assessment and management areas should be revised. For instance, Guadalupe Island should be considered as an independent stock, separate from any of the coastal locations (putative Zone I). Locations in the northern part of the BPC (north of Guerrero Negro, covering San Jerónimo Island, and Faro San José) would constitute a second subpopulation (putative Zone II). All locations from Cedros Island to La Bocana (and Punta Abreojos) would be a third group (putative Zone III). Locations south of San Ignacio Lagoon, which were not sampled in this study, would keep its present classification as Zone IV, until further sampling and analysis are performed.

Nevertheless, since differences are evident in some biological and morphological characteristics, as well as productivity of *H. fulgens* banks along the four administrative zones (Ortíz-Quintanilla & León-Carballo, 1996; Vélez-Arellano, 2016; Guzmán-del Próo & Del Monte-Luna, 2017), the genetic results should be complemented with other techniques (morphological, reproductive and recruitment rates, etc.) to improve management decisions about the resource and help to better delineate the stock units.

## Conclusions

The results of neutral genomic structure do not support the current administrative management areas of green abalone fishery in the BCP. This study showed robust patterns of population structure that should be considered to delineate stock boundaries, assessment, and management actions to ensure fishery resilience. Nevertheless, additional sampling including sites along the whole species distribution range is required to have a better understanding of the spatial genomic diversity. Furthermore, future integration of adaptive variation and environmental and oceanographic modeling will improve the understanding of the biocomplexity of this fishery resource.

## Supplemental Information

10.7717/peerj.9722/supp-1 Supplemental Information 1 Parameter optimization in STACKS.

10.7717/peerj.9722/supp-2 Supplemental Information 2 Number of SNPs retained after filtering steps for green abalone (*Haliotis fulgens*).

10.7717/peerj.9722/supp-3 Supplemental Information 3 Comparison of observed heterozygosity (*Ho*) and allelic richness (*Ar*) using 2170 SNPs among 10 sample sites of green abalone (Haliotis *fulgens*).

10.7717/peerj.9722/supp-4 Supplemental Information 4 Pairwise estimates of FST among 10 sample sites of the green abalone (*Haliotis fulgens*). Significant values are shown in bold after Bonferroni correction for multiple comparisons (*p* < 0.001).

10.7717/peerj.9722/supp-5 Supplemental Information 5 Hierarchical analysis of molecular variance (AMOVA) among 10 sampling sites of *H. fulgens*. AMOVA to examine the partitioning of genetic variance at four hierarchical levels among regional groups of populations identified by the previous analysis, and with different hypothesized structures: (A) administrative zones, (B) two groups based on STRUCTURE results, (C) three groups based on *F*_ST_ results, (D) three groups based on DAPC and UPGMA results, and (E) four groups based on visual division on the DAPC results. Significant values are shown in bold (*p* < 0.05).

10.7717/peerj.9722/supp-6 Supplemental Information 6 Phenogram showing genetic distances among 2,170 SNPs from 10 sampling sites for green abalone (*Haliotis fulgens*). This tree is constructed by the UPGMA method from distance chord. Numbers at the nodes represent the percentage of a group’s occurrence in 5,000 bootstraps. Only those with values higher than 60% are reported. This is an unrooted tree. GI= Guadalupe Island, SJI= San Jerónimo Island, FSJ= Faro San José, CI= Cedros Island, PE= Punta Eugenia, TN= Tortugas North, TS= Tortugas South, AN= Asunción North, AS= Asunción South, Bo= Bocana.

10.7717/peerj.9722/supp-7 Supplemental Information 7 Mantel test with 2-dimensional kernel density estimator. Isolation-by-distance plots of linearized *F*_ST_ values against population pairwise distance for the 10 localities of *H. fulgens* based on 2,170 SNPs among 10 sample sites with a 2-dimensional kernel density estimator. (A) using all the sampling locations, (B) without Guadalupe Island, and (C) using only the central-south locations.

10.7717/peerj.9722/supp-8 Supplemental Information 8 Individual genotypes of 2,170 neutral SNPs from 10 sampling sites for green abalone (*Haliotis fulgens*) along the Baja California Peninsula.

## Additional Information and Declarations

### Field Study Permissions

The following information was supplied relating to field study approvals (i.e., approving body and any reference numbers): Secretaría de Agricultura Ganadería y Desarrollo Rural Pesca y Alimentos (SAGARPA) approved sample collection (permits: PRMN/DGOPA-011/2017; PRMN/DGOPA/016-2018).

### Data Availability

The following information was supplied regarding data availability: The raw genotypes are available in the Supplemental File.

## References

[ref-1] Adamack ET, Gruber B (2014). PopGenReport: simplifying basic population genetic analyses in R. Methods in Ecology and Evolution.

[ref-2] Allendorf FW, Hohenlohe PA, Luikart G (2010). Genomics and the future of conservation genetics. Nature Reviews: Genetics.

[ref-3] Appleyard AS, Carr AN, Elliott GN (2009). Molecular analyses indicate homogenous structure of abalone across morphologically different *Haliotis rubra* collections in South Australia. Journal of Shellfish Research.

[ref-4] Benjamini Y, Hochberg Y (1995). Controlling the false discovery rate: a practical and powerful approach to multiple testing. Journal of the Royal Statistical Society.

[ref-5] Bernardi G (2000). Barriers to gene flow in *Embiotoca jacksoni*, a marine fish lacking a pelagic larval stage. Evolution.

[ref-6] Bernardi G, Talley D (2000). Genetic evidence for limited dispersal in the coastal California killifish, *Fundulus parvipinnis*. Journal of Experimental Marine Biology and Ecology.

[ref-7] Bernatchez L, Wellenreuther M, Araneda C, Ashton DT, Barth JMI, Beacham TD, Maes GE, Martinsohn JT, Miller KM, Naish KA, Ovenden JR, Primmer CR, Suk HY, Therkildsen NO, Withler RE (2017). Harnessing the power of genomics to secure the future of seafood. Trends in Ecology & Evolution.

[ref-8] Briggs JC (1974). Marine zoogeography.

[ref-9] Caddy JF, Mahon R (1995). Reference points for fisheries management. FAO Fisheries Technical Paper.

[ref-10] Catchen MJ, Amores A, Hohenlohe P, Cresko W, Postlethwait HJ (2011). Stacks: building and genotyping loci *de novo* from short-reads sequences. G3: Genes Genomes Genetics.

[ref-11] California Department of Fish and Game Marine Region (2005). Abalone recovery and management plan. https://wildlife.ca.gov/Conservation/Marine/ARMP.

[ref-12] Chelton BD (1982). Large-scale response of the California current to forcing by the wind stress curl. CalCOFI Report.

[ref-13] Danecek P, Auton A, Abecasis G, Albers CA, Banks E, DePristo MA, Handsaker RE, Lunter G, Marth GT, Sherry ST, McVean G, Durbin R, 1000 Genomes Project Analysis Group (2011). The variant call format and VCFtools. Bioinformatics.

[ref-14] Der-Merwe BA, Roodt-Wilding R, Volckaert MAF, D’Amato EM (2011). Historical isolation and hydrodynamically constrained gene flow in declining populations of the South-African abalone, *Haliotis midae*. Conservation Genetics.

[ref-15] DeWoody JA, Avise JC (2000). Microsatellite variation in marine, freshwater and anadromous fishes compared with other animals. Journal of Fish Biology.

[ref-16] Díaz-Viloria N, Cruz-Hernández P, Guzmán-del Proo AS, Pérez-Enríquez R (2009). Genetic connectivity among pink abalone *Haliotis corrugata* populations. Journal of Shellfish Research.

[ref-17] Diario Oficial de la Federación (DOF) (1993). Proyecto de Norma Oficial Mexicana NOM-005-PESC-1993, para regular el aprovechamiento de las poblaciones de las distintas especies de abulón en aguas de jurisdicción federal de la península de Baja California. Diario Oficial de la Federación, Miércoles 1 de septiembre de 1993. http://dof.gob.mx/nota_detalle.php?codigo=4779603&fecha=01/09/1993&print=true.

[ref-18] Diario Oficial de la Federación (DOF) (1994). Aviso por el que se da a conocer el establecimiento de épocas y zonas de veda para la pesca de diferentes especies de la fauna acuática en aguas de jurisdicción federal de los Estados Unidos Mexicanos. Dario Oficial de la Federación, Miércoles 16 de marzo de 1994. http://www.dof.gob.mx/nota_detalle.php?codigo=4678590&fecha=16/03/1994.

[ref-19] Diario Oficial de la Federación (DOF) (2012). Acuerdo por el que se da a conocer la actualización de la Carta Nacional Pesquera, Diario Oficial de la Federación. 24 de Agosto del 2012. Segunda sección. Secretaría de Agricultura Ganadería, Desarrollo Rural, Pesca y Alimentación. México. https://www.dof.gob.mx/nota_detalle.php?codigo=5265388&fecha=24/08/2012.

[ref-20] Diario Oficial de la Federación (DOF) (2018). Acuerdo por el que se da a conocer la actualización de la Carta Nacional Pesquera. Diario Oficial de la Federación, Lunes 11 de junio de. http://dof.gob.mx/nota_detalle.php?codigo=5525712&fecha=11/06/2018.

[ref-21] Dowling AN, Stephen JH, McGarvey R (2004). Assessing population sustainability and response to fishing in terms of aggregation structure for greenlip abalone (*Haliotis laevigata*) fishery management. Canadian Journal of Fisheries and Aquatic Sciences.

[ref-22] Durazo R, Ramírez-Manguilar AM, Miranda LE, Soto-Mardones LA, Gaxiola Castro G, Durazo R (2010). Climatología de variables hidrográficas. Dinámica del ecosistema pelágico frente a Baja California, 1997–2007: Diez años de investigaciones mexicanas de la Corriente de California.

[ref-23] Durazo R (2015). Seasonality of the transitional region of the California current system off Baja California. Journal of Geophysical Research: Oceans.

[ref-24] Epskamp S, Cramer AOJ, Waldorp LJ, Schmittmann VD, Borsboom D (2012). qgraph: network visualizations of relationships in psychometric data. Journal of Statistical Software.

[ref-25] Evanno G, Regnaut S, Goudet J (2005). Detecting the number of clusters of individuals using the software STRUCTURE: a simulation study. Molecular Ecology.

[ref-26] Excoffier L, Lischer HEL (2010). Arlequin suite ver 3.5.2.2: a new series of programs to perform population genetics analyses under Linux and Windows. Molecular Ecology Resources.

[ref-27] Felsenstein J (1989). PHYLIP—phylogeny inference package (Version 3.2). Cladistics.

[ref-28] Foll M, Gaggiotti O (2008). A genome-scan method to identify selected loci appropriate for both dominant and codominant markers: a Bayesian perspective. Genetics.

[ref-29] Foll M (2012). BayeScan v2.1 user manual. Ecology.

[ref-30] Frank KT, Brickman D (2000). Allee effect and compensatory population dynamics within a stock complex. Canadian Journal of Fisheries and Aquatic Sciences.

[ref-31] Funk CW, McKay KJ, Hohenlohe AP, Allendorf WF (2012). Harnessing genomics for delineating conservation units. Trends in Ecology and Evolution.

[ref-32] Gilbey J, Cauwelier E, Coulson WM, Stradmeyer L, Sampayo NJ, Armstrong A, Verspoor E, Corrigan L, Shelley J, Middlemas S (2016). Accuracy of assignment of Atlantic salmon (*Salmo salar* L.) to rivers and regions in Sctotland and Northeast England based on single nucleotide polymorphism (SNP) markers. PLOS ONE.

[ref-33] Goudet J, Jombart T (2015). https://CRAN.R-project.org/package=hierfstat.

[ref-34] Grewe PM, Feutry P, Hill PL, Gunasekera RM, Schaefer KM, Itano DG, Fuller DW, Foster SD, Davies CR (2015). Evidence of discrete yellowfin tuna (*Thunnus albacares*) populations demands rethink of management for this globally important resource. Scientific Reports.

[ref-35] Gruenthal KM, Witting DA, Ford T, Neuman MJ, Williams JP, Pondella DJ, Bird A, Caruso N, Hyde JR, Seeb LW, Larson WA (2013). Development and application of genomic tools to the restoration of green abalone in southern California. Conservation Genetics.

[ref-36] Gutiérrez-González JL, Cruz-Hernández P, Del Río-Portilla MA, Pérez-Enríquez R (2007). Genetic structure of green abalone *Haliotis fulgens* populations of Baja California, México. Journal of Shellfish Research.

[ref-38] Guzmán-del Próo SA, Shepard SA, Tegner MJ, Guzmán-del Próo SA (1992). A review of the biology of abalone and its fishery in Mexico. Abalone of the World. Biology, Fisheries and Culture.

[ref-39] Guzmán-del Proo SA, Salinas F, Zaytsev O, Belmar-Pérez J, Carrillo-Laguna J (2000). Potential dispersion of reproductive products and larval stages of abalone *Haliotis* spp. As a function of the hydrodynamics of Bahia Tortugas, México. Journal of Shellfish Research.

[ref-40] Guzmán-del Próo SA, Carreón-Palau L, Belmar-Pérez J, Carrillo-Laguna J, Fragoso R (2003). Effects os the ‘El Niño’ event on the recruitment of benthic invertebrates in Bahía Tortugas, Baja California Sur. Geofísica Internacional.

[ref-41] Guzmán-del Próo SA, Del Monte-Luna P (2017). Abalone reef productivity and the problem of scale in the management of the Mexican abalone fishery. Ocean & Costal Management.

[ref-42] Hamm DE, Burton RS (2000). Population genetics of black abalone, *Haliotis cracherodii*, along the central California coast. Journal of Experimental Marine Biology and Ecology.

[ref-43] Hammer-Hansen J, Therkildsen NO, Pujolar JM (2014). Population genomics of marine fishes: next-generation prospects and challenges. Biological Bulletin.

[ref-44] Haupt AJ, Micheli F, Palumbi SR (2013). Dispersal at a snail’s pace: historical processes affect contemporary genetic structure in the exploited wavy top snail (*Megastraea undosa*). Journal of Heredity.

[ref-45] Hauser L, Carvalho GR (2008). Paradigm shifts in marine fisheries genetics: ugly hypotheses slain by beautiful facts. Fish and Fisheries.

[ref-46] Hedgecock D, Launey S, Pudovkin AI, Naciri Y, Lapègue S, Bonhomme F (2007). Small effective number of parents (Nb) inferred for a naturally spawned cohort of juvenile European flat oysters *Ostrea edulis*. Marine Biology.

[ref-47] Hewitt RP (1981). Eddies and speciation in the California Current. CalCOFI Report.

[ref-48] Hilborn R, Quinn TP, Schindler DE, Rogers DE (2003). Biocomplexity and fisheries sustainability. Proceedings of the National Academy of Sciences.

[ref-49] Hilborn R, Walters CJ (1992). Quantitative fisheries stock assessment: choice, dynamics and uncertainty.

[ref-50] Hobday JA, Tegner JM, Haaker LP (2001). Over-explotation of a broadcast spawning marine invertebrate: decline of the white abalone. Reviews in Fish Biology and Fisheries.

[ref-51] Huang D, Bernardi G (2001). Disjunct Sea of Cortez-Pacific Ocean *Gillichthys mirabilis* populations and evolutionary origin of their Sea of Cortez endemic relative, *Gillichthys seta*. Marine Biology.

[ref-52] Jombart T, Devillard S, Balloux F (2010). Discriminant analysis of principal components: a new method for the analysis of genetically structured populations. BMC Genetics.

[ref-53] Kamvar NZ, Tabima FJ, Grünwald JN (2014). Poppr: an R package for genetic analysis of populations with clonal, partially clonal, and/or sexual reproduction. PeerJ.

[ref-54] Keenan K, McGinnity P, Cross TF, Crozier WW, Prodöhl PA (2013). DiveRsity: an R package for the estimation and exploration of population genetics parameters and their associated errors. Methods in Ecology and Evolution.

[ref-55] Kenchington EL, Sinclair M, Valdimarson G (2003). The effects of fishing on species and genetic diversity. Responsible Fisheries in the Marine Ecosystem.

[ref-56] Konietschke F, Noguchi K, Rubarth K (2019). Package ‘nparcomp’ multiple comparisons and simultaneous confidence intervals. https://cran.r-project.org/package=nparcomp.

[ref-57] Laconcha U, Iriondo M, Arrizabalaga H, Manzano C, Markaide P, Montes I, Zarraonaindia I, Velado I, Bilbao E, Goñi N, Santiago J, Domingo A, Karakulak S, Oray I, Estonba A (2015). New nuclear SNP markers unravel the genetic structure and effective population size of albacore tuna (*Thunnus alalonga*). PLOS ONE.

[ref-58] Laikre L, Palm S, Ryman N (2005). Genetic population structure of fishes: implications for coastal zone management. AMBIO: A Journal of the Human Environment.

[ref-59] Lavaniegos BE (2014). Pelagic amphipod assemblage associated with subarctic water off the West Coast of the Baja California Peninsula. Journal of Marine Systems.

[ref-60] Leighton LD (2000). The biology and culture of the California abalones.

[ref-61] Lischer HEL, Excoffier L (2012). PGDSpider: an automated data conversion tool for connecting population genetics and genomics programs. Bioinformatics.

[ref-62] Lluch-Belda (1999). Coastal upwelling in the eastern Gulf of California. Oceanologica Acta.

[ref-63] Lluch-Belda D, Elorduy-Garay J, Lluch-Cota SE, Ponce-Díaz G (2000). BAC Centros de Actividad Biológica del Pacífico Mexicano.

[ref-64] Luikart G, England PR, Tallmon D, Jordan S, Taberlet P (2003). The power and promise of population genomics: from genotyping to genome typing. Nature Reviews: Genetics.

[ref-65] Lynn RJ, Simpson JJ (1987). The California current system: the seasonal variability of its physical characteristics. Journal of Geophysical Research.

[ref-66] Mantel N (1967). The detection of disease clustering and a generalized regression approach. Cancer Research.

[ref-98] Meirmans PG (2012). The trouble with isolation by distance. Molecular Ecology.

[ref-67] Morgan L, Shepherd SA, Kritzer PJ, Sale FP (2006). Population and spatial structure of two common temperate reef herbivores. Abalone and sea urchins. In Marine Metapopulations.

[ref-68] Mullins RB, McKeown NJ, Sauer WHH, Shaw PW, Grant WS (2018). Genomic analysis reveals multiple mismatches between biological and management units in yellowfin tuna (*Thunnus albacares*). ICES Journal of Marine Science.

[ref-69] Munguía-Vega A, Sáenz-Arroyo A, Greenley AP, Espinoza-Montes JA, Palumbi SR, Rossetto M, Micheli F (2015). Marine reserves help preserve genetic diversity after impacts derived from climate variability: lessons from the pink abalone in Baja California. Global Ecology and Conservation.

[ref-70] Muñiz-Salazar R, Talbot LS, Sage KG, Ward HD, Pasini-Cabello A (2005). Population genetics structure of annual and perennial populations of *Zoostera marina* L. along the Pacific coast of Baja California and the Gulf of California. Molecular Ecology.

[ref-71] Nei M (1973). Analysis of gene diversity in subdivided populations. Proceedings of the National Academy of Sciences.

[ref-72] Paris JR, Stevens JR, Catchen JM (2017). Lost in parameter space: a road map for STACKS. Methods in Ecology and Evolution.

[ref-99] Ortíz-Quintanilla M, León-Carballo G (1996). La pesquería del abulón. Pesquerías relevantes de México XXX aniversario del INP (1962–1992).

[ref-73] Ortiz-Quintanilla M, Leon-Carballo G (1988). Los recursos pesqueros de México y sus perspectivas, recurso abulón *Haliotis* spp. Secretaria de Pesca-INP.

[ref-74] Page RDM (1996). TREEVIEW: an application to display phylogenetic trees on personal computers. Computer Applications in the Biosciences.

[ref-75] Pecoraro C, Babbucci M, Franch R, Rico C, Papetti C, Chassot E, Bodin N, Cariani A, Bargelloni L, Tini F (2018). The population genomics of yellowfin tuna (*Thunnus albacares*) at global geographic scale challenges current stock delineation. Scientific Reports.

[ref-76] Peterson BK, Weber JN, Kay EH, Fisher HS, Hoekstra HE (2012). Double digest RADseq: an inexpensive method for De Novo SNP discovery and genotyping in model and non-model species. PLOS ONE.

[ref-77] Pitchard JK, Stephens M, Donnelly P (2000). Inference of population structure using multilocus genotype data. Genetics.

[ref-78] R core Team (2014). R: a language and environment for statistical computing.

[ref-79] Reiss H, Hoarau G, Dickey-Collas M, Wolff WJ (2009). Genetic population structure of marine fish: mismatch between biological and fisheries management units. Fish and Fisheries.

[ref-80] Rochette NV, Catchen JM (2017). Deriving genotypes from RAD-seq short-read data using stacks. Nature Protocols.

[ref-81] Rousset F (1997). Genetic differentiation and estimation of gene flow from F-statistics under isolation by distance. Genetics.

[ref-82] Sambrook J, Fritsch EF, Maniatis T (1989). Molecular cloning: a laboratory manual.

[ref-83] Selkoe KA, Toonen RJ (2006). Microsatellites for ecologists: a practical guide to using and evaluating microsatellite markers. Ecology Letters.

[ref-96] Shepherd SA, Guzman-del Proo SA, Turrubiates J, Belmar J, Baker LJ, Sluczanowski PR (1991). Growth, size at sexual maturity, and egg-per-recruit analysis of the abalone Haliotis fulgens in Baja California. The Veliger.

[ref-84] Shepherd AS, Brown DL (1993). What is in abalone stock: implications for the role of refugia in conservations. Canadian Journal of Fisheries and Aquatics Sciences.

[ref-85] Sierra-Rodríguez P, Muciño-Díaz M, Gutiérrez-González JL, Turrubiates-Morales J (2006). Sustentabilidad y pesca responsable en México: Evaluación y Manejo.

[ref-86] Sundqvist L, Keenan K, Zackrisson M, Prodöhl P, Kleinhans D (2016). Directional genetic differentiation and relative migration. Ecology and Evolution.

[ref-97] Tegner MJ, Butler RA (1985). Drift-tube study of the dispersal potential of green abalone (Haliotis fulgens) larvae in the southern California Bight: implications for recovery of depleted populations. Marine Ecology—Progress Series.

[ref-87] Terry A, Bucciarelli G, Bernardi G (2000). Restricted gene flow and incipient speciation in disjunct Pacific Ocean and Sea of Cortez populations of a reef fish species, *Girella nigricans*. Evolution.

[ref-89] Vélez-Arellano NM (2016). Tácticas reproductivas de *Haliotis fulgens* Philippi, 1845 y *Haliotis corrugata* W. Wood, 1828 (Gastropoda: Archeograstropoda) en zonas de pesca en la costa occidental de Baja California Sur.

[ref-90] Vélez-Arellano NM, García-Domínguez FA, Lluch-Cota DB, Gutiérrez-González JL, Salcido-Guevara LA, Sanchez-Cardenas R (2017). Morphophysiological indices of the green abalone *Haliotis fulgens* Philippi, 1845 at Mexican Ocean Pacific coast. Turkish Journal of Fihseries and Aquatic Sciences.

[ref-91] Vélez-Arellano NM, Valenzuela-Quiñonez F, García-Domínguez FA, Lluch-Cota DB, Gutiérrez-González JL, Martínez-Rincón RO (2020). Long-term analysis on the spawning activity of green (*Haliotis fulgens*) and pink (*Haliotis corrugata*) abalone along the central west coast of Baja California. Fisheries Research.

[ref-95] Venables WN, Ripley BD (2002). Modern applied statistics with S.

[ref-92] Weir BS, Cockerham CC (1984). Estimating F-statistics for the analysis of population structure. Evolution.

[ref-93] Withler ER, Campbell A, Li S, Miller MK, Brouwer D, Lucas GB (2001). High levels of genetic variation in northern abalone *Haliotis kamtschatkana* of British Columbia. Fisheries and Oceans Science.

[ref-94] Zúñiga G, Guzmán-del Proó SA, Cisneros R, Rodríguez G (2000). Genetic population analysis of abalone *Haliotis fulgens* (Mollusca: Gastropoda) in Baja California. Mexico Journal of Shellfish Research.

