# Supplementary material for: Population genomics reveals a mismatch between management and biological units in green abalone (Haliotis fulgens)
Source: PeerJ. 2020 Aug 19;8:e9722. doi: 10.7717/peerj.9722 (PMC7443094; doi:10.7717/peerj.9722)
Supplement: Supplemental Information 1 [file peerj-08-9722-s001.docx]

**S1. Parameter optimisation in STACKS**

Figure A) Combinations of the main parameters using the denovo_map.pl pipeline in STACKS. Minimum stack depth (-m), maximum number of nucleotide mismatches between stacks (-M) and maximum number of mismatches between sample loci (-n).


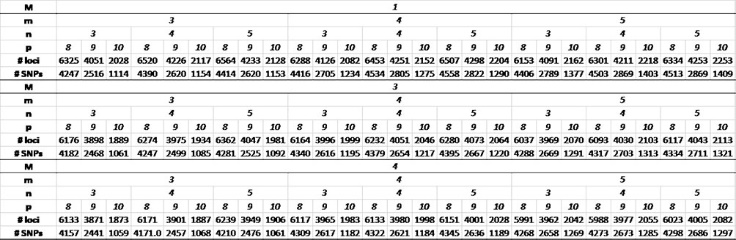


Reads were assembled *de novo* using the *denovo_map.pl* program in STACKS pipeline. This program has 3 key parameters (m, M, and n), which frequently have a significant effect on the building and quality of the resulting loci. The minimum number of raw reads required to form a stack (or putative allele) -***m*,** the number of mismatches allowed between stacks to merge them into a putative locus (within individuals) -***M*,** and the number of mismatches allowed between stacks (between individuals) ***-n***.

We ran *denovo_map.pl* several times, varying just one parameter with each parse of the program. We also tested for different values of ***-p* (**from 8 to 10) on the program populations, which performs the filtering of the loci and population genetics analysis (Figure A).

We found that as higher values ​​of ***-m*** and ***-M*** are used, the average depth increases and there is no apparent effect of ***-n***. Besides, there is no large difference between the depth values when M increases from 3 to 4 (Figure B).


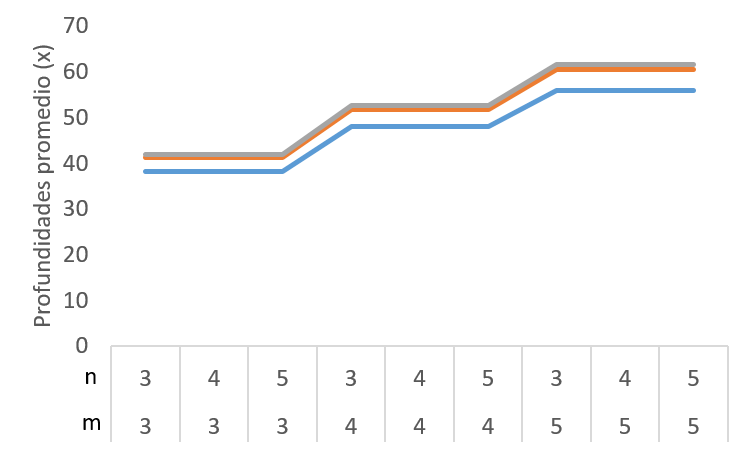


M1

M3

M4

Figure B. The effect of ***-m***, ***-M***, and ***-n*** parameters on the mean depth (coverage).

When we analyzed the number of loci and SNPs, we observed that the number of assembled loci decreases as ***m*** increases (Figure C). Furthermore, there is a slight increase in the number of SNPs as ***n*** increases, and once again, the effect of -***M*** on the number of loci and SNPs becomes imperceptible (Figure D).


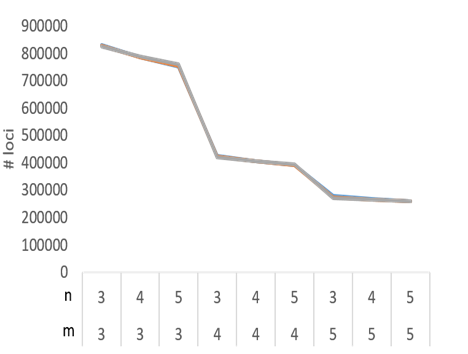


Figure C. The effect of ***-m***, ***-M***, and ***-n*** on the number of loci retained.


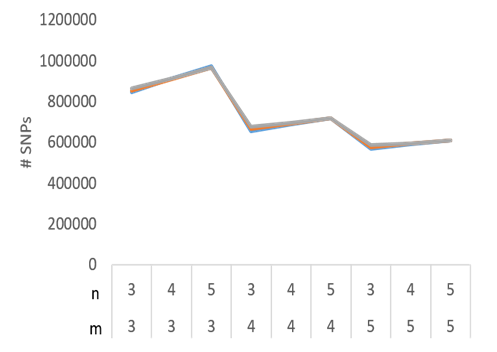


Figure D. The effect of ***-m***, ***-M***, and ***-n*** on the number of SNPs retained.

When the ***-M*** parameter is set too low, alleles from the same locus will not collapse. On the other hand, if it is set too high, paralogous or repetitive loci will incorrectly merge together. Given that the effect of -***M*** on the depth, the number of loci and SNPs, becomes imperceptible when ***-M*** increases from 3 to 4 we decided to keep this value on 3.

For each parameter combination, we filtered the raw results of the pipeline using the *populations* module, keeping only loci shared by at least 80% of samples (-r 0.80).

The parameter ***-m*** was decided to set it on 5 because we found that the number of loci and SNPs is higher with smaller -***m*** values (Figures C and D) when we tested for different values of ***-p***. These loci were not shared throughout all the locations, so they are lost and the difference becomes imperceptible when the ***population*** program is run (Figures H and I). We also observed a large drop in the number of loci assembled between ***-m*** 3 and ***-m*** 4, but between ***-m*** 4 and ***-m*** 5 the number of loci tends to stabilize (Figure C).


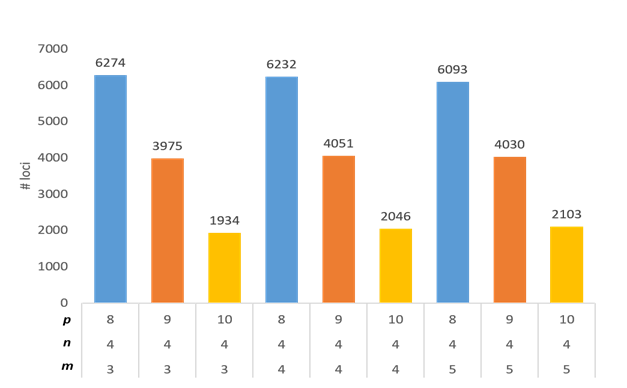


Figure E. The effect of ***-m***, ***-n***, and -***p*** on the number of loci retained.


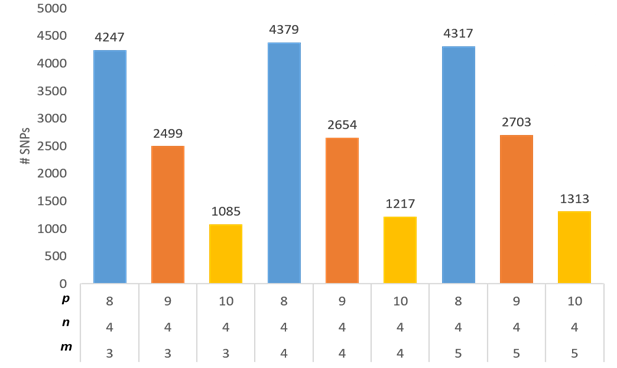


Figure F. The effect of ***-m***, ***-n***, and -***p*** on the number of SNPs retained.

What stands out is a dramatical decrease in the number of loci and SNPs as the ***-p*** value increases, regardless of the value of ***-m*** and / or ***-n***.

Since the depth, number of SNPs, and loci were not that affected with the ***-n*** parameter, we decided to follow the rule proposed by Paris et al. (2017) (***M***+1).

As a result, the data set obtained with ***-M*** 3 ***-m*** 5 ***-n*** 4 recovered a greater number of SNPs in almost all the tests of ***-p***, which is why we decided to use those data set (with different values of ***-p***) to further investigate the effect on the population structure.

Before performing genetic population structure analysis, we used the Vcftools program to evaluate the percentage of missing genotypes for each individual with the SNPs obtained for ***-p*** 8, 9 and 10.

We observed that, despite recovering a larger number of markers using a smaller value of ***-p*** of (8) the value of missing genotypes is greater than 30% which decreases to 20% and 10% with a ***-p*** of 9 and 10 respectively.

Finally, it was decided to evaluate if the population structure remained constant regardless of the set of SNPs used. Given that no greater differences were found in the structuring patterns with any of the data sets, we used the parameters and the data set with the highest number of SNPs with the least missing data values (M 3, m 5, n 4, p 10, r 0.8).
