# Supplementary material for: Population genomics reveals a mismatch between management and biological units in green abalone (Haliotis fulgens)
Source: PeerJ. 2020 Aug 19;8:e9722. doi: 10.7717/peerj.9722 (PMC7443094; doi:10.7717/peerj.9722)
Supplement: Supplemental Information 2 [file peerj-08-9722-s002.docx]

| **Filtering step** | **# SNPs retained** |
| --- | --- |
| **STACKS** |  |
| ***denovo_map*** (***M***3***m***5***n***4) | 353,332 |
| ***populations*** present in at least 80% of the individuals at each sampling site (***-r*** 0.08) and in 90% of localities (***-p*** 9), with a minor allele frequency higher than 0.05 | 2,216 |
|  |  |
| **Outlier detection** |  |
| **Arlequin** (45 loci) |  |
| **Bayescan** (5 loci) |  |
| **Identified by both methods** (5 loci) | 2,171 |
|  |  |
| **Hardy-Weinberg** **Equilibrium** (>50% of localities) (1 locus) |  |
| **Putative neutral data set** | 2,170 |

**S2.** Number of SNPs retained after filtering steps for green abalone (*Haliotis fulgens*).
