# Supplementary material for: Population genomics reveals a mismatch between management and biological units in green abalone (Haliotis fulgens)
Source: PeerJ. 2020 Aug 19;8:e9722. doi: 10.7717/peerj.9722 (PMC7443094; doi:10.7717/peerj.9722)
Supplement: Supplemental Information 3 [file peerj-08-9722-s003.docx]

**S3.**

Comparison of observed heterozygosity (*Ho*) using 2170 SNPs among 10 sample sites of green abalone (*Haliotis fulgens*) using the **Gao test**. *p*-adjusted values of non-parametric multiple comparisons (lower triangle) and alpha values for each comparison (upper triangle) of Significant comparisons are shown in bold (*p* < alpha value).

| *Ho* | GI | SJI | FSJ | CI | PE | TN | TS | AN | AS | Bo |
| --- | --- | --- | --- | --- | --- | --- | --- | --- | --- | --- |
| GI | * | 0.010 | 0.010 | 0.035 | 0.040 | 0.015 | 0.050 | 0.025 | 0.020 | 0.030 |
| SJI | 0.772 | * | 0.015 | 0.040 | 0.050 | 0.020 | 0.050 | 0.030 | 0.025 | 0.035 |
| FSJ | 0.228 | 0.059 | * | 0.030 | 0.035 | 0.010 | 0.040 | 0.020 | 0.015 | 0.025 |
| CI | 0.035 | **0.027** | 0.814 | * | 0.010 | 0.025 | 0.015 | 0.015 | 0.020 | 0.010 |
| PE | **0.006** | **0.005** | 0.588 | 0.645 | * | 0.030 | 0.010 | 0.020 | 0.025 | 0.030 |
| TN | 0.027 | 0.023 | 0.224 | 0.980 | 0.971 | * | 0.035 | 0.015 | 0.010 | 0.020 |
| TS | **0.001** | **0.001** | 0.319 | 0.535 | 0.862 | 0.849 | * | 0.025 | 0.030 | 0.020 |
| AN | 0.038 | 0.066 | 0.658 | 0.944 | 0.933 | 0.786 | 0.778 | * | 0.010 | 0.010 |
| AS | 0.031 | > 0.025 | 0.579 | 0.925 | 0.893 | 0.650 | 0.773 | 0.665 | * | 0.015 |
| Bo | 0.046 | 0.046 | 0.862 | 0.789 | 0.823 | 1.000 | 0.762 | 0.846 | 0.949 | * |

GI**=** Guadalupe Island, SJI= San Jerónimo Island, FSJ**=** Faro San José, CI**=** Cedros Island, PE= Punta Eugenia, TN= Tortugas North, TS**=** Tortugas South, AN= Asunción North, AS= Asunción South, and Bo= Bocana.

Comparison of Allelic richness (*Ar*) using 2170 SNPs among 10 sample sites of green abalone (*Haliotis fulgens*) using the **Gao test**. *p*-adjusted values of non-parametric multiple comparisons (lower triangle) and alpha values for each comparison (upper triangle). Significant comparisons are shown in bold (*p* < alpha value).

| *Ar* | GI | SJI | FSJ | CI | PE | TN | TS | AN | AS | Bo |
| --- | --- | --- | --- | --- | --- | --- | --- | --- | --- | --- |
| GI | * | 0.0153 | 0.05 | 0.0102 | 0.0353 | 0.0402 | 0.0303 | 0.05 | 0.0253 | 0.0203 |
| SJI | 0.2817 | * | 0.0402 | 0.0102 | 0.0253 | 0.0303 | 0.0203 | 0.0353 | 0.0153 | 0.0102 |
| FSJ | **0.0001** | 0.0876 | * | 0.05 | 0.0203 | 0.0153 | 0.0253 | 0.0102 | 0.0303 | 0.0353 |
| CI | 0.0957 | 0.8338 | 0.0664 | * | 0.0303 | 0.0353 | 0.0253 | 0.0402 | 0.0203 | 0.0153 |
| PE | 0.0503 | 0.8542 | 0.2625 | 0.933 | * | 0.0102 | 0.0102 | 0.0153 | 0.0153 | 0.0203 |
| TN | **0.0062** | 0.5187 | 0.3281 | 0.5208 | 0.4019 | * | 0.0153 | 0.0102 | 0.0203 | 0.0253 |
| TS | 0.0814 | 0.8888 | 0.1367 | 0.9517 | 0.6771 | 0.5307 | * | 0.0203 | 0.0102 | 0.0153 |
| AN | **0.0015** | 0.3205 | 0.5028 | 0.2915 | 0.3052 | 0.316 | 0.3483 | * | 0.0253 | 0.0303 |
| AS | 0.1735 | 0.952 | 0.1115 | 0.9979 | 0.7839 | 0.4574 | 0.7805 | 0.2532 | * | 0.0102 |
| Bo | 0.2464 | 0.9849 | 0.0663 | 0.9878 | 0.7598 | 0.3451 | 0.8192 | 0.1646 | 0.7675 | * |

GI**=** Guadalupe Island, SJI= San Jerónimo Island, FSJ**=** Faro San José, CI**=** Cedros Island, PE= Punta Eugenia, TN= Tortugas North, TS**=** Tortugas South, AN= Asunción North, AS= Asunción South, and Bo= Bocana.
