# Supplementary material for: Population genomics reveals a mismatch between management and biological units in green abalone (Haliotis fulgens)
Source: PeerJ. 2020 Aug 19;8:e9722. doi: 10.7717/peerj.9722 (PMC7443094; doi:10.7717/peerj.9722)
Supplement: Supplemental Information 4 — Significant values are shown in bold after Bonferroni correction for multiple comparisons (p < 0.001). [file peerj-08-9722-s004.docx]

**S4.** Pairwise estimates of *F_ST_* among 10 sample sites of the green abalone (*Haliotis fulgens*). Significant values are shown in bold after Bonferroni correction for multiple comparisons (*p* < 0.001).

|  | GI | SJI | FSJ | CI | PE | TN | | TS | | AN | | AS | | Bo | |
| --- | --- | --- | --- | --- | --- | --- | --- | --- | --- | --- | --- | --- | --- | --- | --- |
| GI | 0.000 |  |  |  |  | |  | |  | |  | |  | |  |
| SJI | **0.018** | 0.000 |  |  |  | |  | |  | |  | |  | |  |
| FSJ | **0.021** | 0.007 | 0.000 |  |  | |  | |  | |  | |  | |  |
| CI | **0.015** | **0.007** | 0.004 | 0.000 |  | |  | |  | |  | |  | |  |
| PE | **0.017** | **0.008** | 0.004 | -0.001 | 0.000 | |  | |  | |  | |  | |  |
| TN | **0.019** | **0.010** | 0.006 | 0.001 | -0.001 | | 0.000 | |  | |  | |  | |  |
| TS | **0.018** | **0.008** | **0.006** | -0.001 | 0.000 | | 0.003 | | 0.000 | |  | |  | |  |
| AN | **0.017** | **0.011** | 0.005 | -0.001 | -0.001 | | 0.000 | | 0.002 | | 0.000 | |  | |  |
| AS | **0.017** | **0.012** | 0.004 | 0.002 | 0.000 | | 0.003 | | 0.001 | | 0.003 | | 0.000 | |  |
| Bo | **0.016** | **0.010** | 0.004 | -0.002 | -0.002 | | 0.001 | | 0.001 | | 0.001 | | 0.002 | | 0.000 |

GI**=** Guadalupe Island, SJI= San Jerónimo Island, FSJ**=** Faro San José, CI**=** Cedros Island, PE= Punta Eugenia, TN= Tortugas North, TS**=** Tortugas South, AN= Asunción North, AS= Asunción South, and Bo= Bocana.
