# Supplementary material for: Population genomics reveals a mismatch between management and biological units in green abalone (Haliotis fulgens)
Source: PeerJ. 2020 Aug 19;8:e9722. doi: 10.7717/peerj.9722 (PMC7443094; doi:10.7717/peerj.9722)
Supplement: Supplemental Information 5 — AMOVA to examine the partitioning of genetic variance at four hierarchical levels among regional groups of populations identified by the previous analysis, and with different hypothesized structures: (A) administrative zones, (B) two groups based on STRUCTURE results, (C) three groups based on FST results, (D) three groups based on DAPC and UPGMA results, and (E) four groups based on visual division on the DAPC results. Significant values are shown in bold (p < 0.05). [file peerj-08-9722-s005.docx]

**S5. Hierarchical analysis of molecular variance (AMOVA) among 10 sampling sites of *H. fulgens*.** AMOVA to examine the partitioning of genetic variance at four hierarchical levels among regional groups of populations identified by previous analysis, and with different hypothesized structures: **A)** administrative zones, **B)** two groups based on STRUCTURE results, **C)** three groups based on *F_ST_* results, **D)** three groups based on DAPC and UPGMA results, and **E)** four groups based on visual division on the DAPC results. Significant values are shown in bold (*p* < 0.05).

|  | **d.f** | **Fixation index** | **Variation (%)** | ***p-*value** |
| --- | --- | --- | --- | --- |
| **A) Administrative zones** |  |  |  |  |
| Among groups (***F_CT_***) | 2 | 0.0003 | 0.03 | 0.297 |
| Among populations within groups (***F_SC_***) | 7 | 0.0055 | 0.55 | **0.000** |
| Among individuals within populations (***F_IS_***) | 165 | 0.0041 | 0.41 | 0.387 |
| Within individuals (***F_IT_***) | 175 | 0.0099 | 99.01 | 0.243 |
| **B) 2 Groups (GI, all other localities) based on STRUCTURE results** | | | | |
| Among groups (***F_CT_***) | 1 | 0.0148 | 1.48 | 0.096 |
| Among populations within groups (***F_SC_***) | 8 | 0.0023 | 0.23 | **0.000** |
| Among individuals within populations (***F_IS_***) | 165 | 0.0041 | 0.41 | 0.380 |
| Within individuals (***F_IT_***) | 175 | 0.0212 | 97.88 | 0.240 |
| **C) 3 Groups (GI, SJI, all other localities) based on *FST* results** | | | | |
| Among groups (***F_CT_***) | 2 | 0.0126 | 1.26 | **0.0230** |
| Among populations within groups (***F_SC_***) | 7 | 0.0011 | 0.11 | **0.0241** |
| Among individuals within populations (***F_IS_***) | 165 | 0.0041 | 0.41 | 0.3745 |
| Within individuals (***F_IT_***) | 175 | 0.0178 | 98.22 | 0.2423 |
| **D) 3 Groups (GI, SJI+ FSJ, all other localities based on DAPC and UPGMA results** | | | | |
| Among groups (***F_CT_***) | 2 | 0.0104 | 1.04 | **0.0059** |
| Among populations within groups (***F_SC_***) | 7 | 0.0009 | 0.09 | **0.0274** |
| Among individuals within populations (***F_IS_***) | 165 | 0.0041 | 0.41 | 0.3979 |
| Within individuals (***F_IT_***) | 175 | 0.0155 | 98.45 | 0.2454 |
| **E) 4 Groups (GI, SJI, FSJ, all other localities based** **on visual division on the DAPC results** | | | | |
| Among groups (***F_CT_***) | 3 | 0.0112 | 1.12 | **0.0088** |
| Among populations within groups (***F_SC_***) | 6 | 0.0005 | 0.05 | 0.2227 |
| Among individuals within populations (***F_IS_***) | 165 | 0.0041 | 0.41 | 0.3879 |
| Within individuals (***F_IT_***) | 175 | 0.0158 | 98.42 | 0.2500 |
|  |  |  |  |  |
