# Supplementary material for: Population genomics reveals a mismatch between management and biological units in green abalone (Haliotis fulgens)
Source: PeerJ. 2020 Aug 19;8:e9722. doi: 10.7717/peerj.9722 (PMC7443094; doi:10.7717/peerj.9722)
Supplement: Supplemental Information 6 — This tree is constructed by the UPGMA method from distance chord. Numbers at the nodes represent the percentage of a group’s occurrence in 5,000 bootstraps. Only those with values higher than 60% are reported. This is an unrooted tree. GI= Guadalupe Island, SJI= San Jerónimo Island, FSJ= Faro San José, CI= Cedros Island, PE= Punta Eugenia, TN= Tortugas North, TS= Tortugas South, AN= Asunción North, AS= Asunción South, Bo= Bocana. [file peerj-08-9722-s006.docx]

 **S6. Phenogram showing genetic distances among 1,772 SNPs from 10 sampling sites of H. fulgens.** This tree is constructed by the UPGMA method from distance chord. Numbers at the nodes represent the percentage of a group’s occurrence in 5,000 bootstraps. Only those with values higher than 60% are reported. This is an unrooted tree. GI**=** Guadalupe Island, SJI= San Jerónimo Island, FSJ**=** Faro San José, CI**=** Cedros Island, PE= Punta Eugenia, TN= Tortugas North, TS**=** Tortugas South, AN= Asunción North, AS= Asunción South, Bo= Bocana.
