# Supplementary material for: Population genomics reveals a mismatch between management and biological units in green abalone (Haliotis fulgens)
Source: PeerJ. 2020 Aug 19;8:e9722. doi: 10.7717/peerj.9722 (PMC7443094; doi:10.7717/peerj.9722)

**S7. Mantel test with 2-dimensional kernel density estimator.** Isolation-by-distance (IBD) plots of linearized *F_ST_* values against population pairwise distance for the 10 localities of *H. fulgens* based on 2,170 SNPs among 10 sample sites with a 2-dimensional kernel density estimator. A) using all the sampling locations, B) without Guadalupe Island, and C) using only the central-south locations.


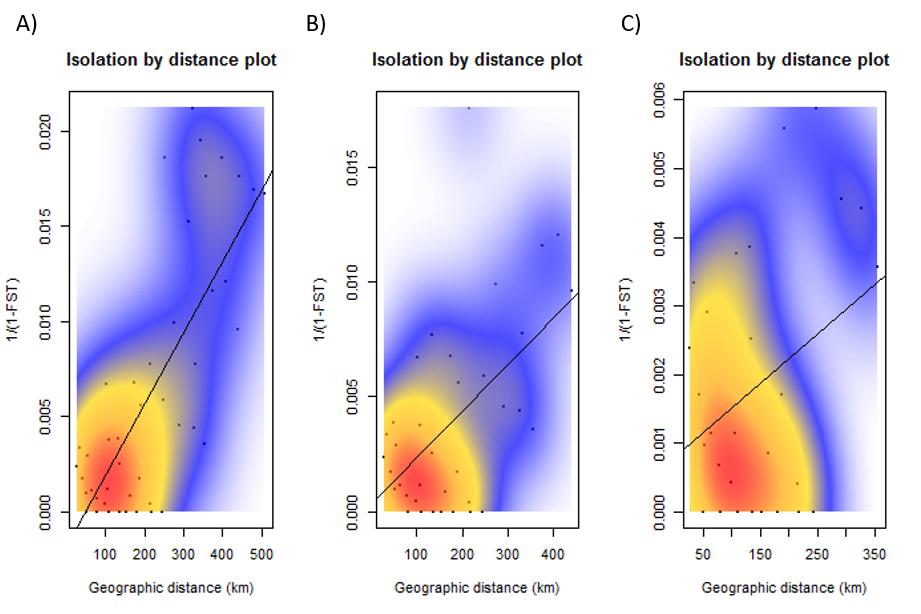

Supplement: Supplemental Information 7 — Isolation-by-distance plots of linearized FST values against population pairwise distance for the 10 localities of H. fulgens based on 2,170 SNPs among 10 sample sites with a 2-dimensional kernel density estimator. (A) using all the sampling locations, (B) without Guadalupe Island, and (C) using only the central-south locations. [file peerj-08-9722-s007.docx]
